# Species of anaerobic Gram-negative bacilli involved in abscesses localized in the fascial spaces of the head and neck

**DOI:** 10.1186/1471-2334-14-S7-P64

**Published:** 2014-10-15

**Authors:** Adrian Băncescu, Andreea Didilescu, Gabriela Băncescu

**Affiliations:** 1 Carol Davila University of Medicine and Pharmacy, Bucharest, Romania

## Background

In general, the oro-maxillo-facial infections, including the abscesses in the head and neck spaces, are mixed infections, involving anaerobic bacteria in association with facultative anaerobic microorganisms, with the predominance of the first ones. Since most anaerobes are fastidious bacteria, their isolation and identification are not routinely performed by the clinical laboratory, being considered too time-consuming. The aim of this study was to identify at species level a collection of 44 anaerobic Gram-negative bacilli strains (isolated from pus samples collected from patients with abscesses in the spaces of the head and neck presented to the Clinical Hospital of Oro-Maxillo-Facial Surgery - Bucharest, during 2011-2012), stored in ultrafreezer at the laboratory of Microbiology Chair of the Faculty of Dentistry, University of Medicine and Pharmacy “Carol Davila” - Bucharest.

## Methods

The 44 strains were presumptively identified at genus level by the conventional diagnostic methods, based on: colony morphology (pigmentation and fluorescence), bile sensitivity test and sensitivity to special-potency antibiotic disks (erythromycin, rifampicin, colistin sulphate, penicillin G, kanamycin and vancomycin), tested by using the MAST ID MID8 ANAEROBE ID RING (MAST Group Ltd., UK). Identification at the species level was done by the Rapid ID 32 A system (BioMérieux, France).

## Results

The anaerobic Gram-negative bacilli strains investigated in this study were included either in *Prevotella* or *Fusobacterium* genus. A number of 33 isolates were identified as *Prevotella* and belonged to the following species: *P. melaninogenica* - 13 strains, *P. oralis* - 7 strains, *P. intermedia* - 5 strains, *P. buccae* - 5 strains and *P. denticola* - 3 strains. The rest of the isolates were identified as *Fusobacterium nucleatum*.

## Conclusion

The *Prevotella* species represented two-thirds of all anaerobic Gram-negative rods isolates. *P. melaninogenica* was the predominating species and was closely followed by *F. nucleatum*. The identification at species level of the clinically significant isolates from oro-maxillo-facial infections might be important for further investigation on the host-pathogen interactions.

